# Editorial: Mitigating agricultural greenhouse gas emissions through bio-inputs and innovative practices

**DOI:** 10.3389/fpls.2026.1967459

**Published:** 2026-09-04

**Authors:** Virgilio Gavicho Uarrota, Marcelo Maraschin, Bente Foereid

**Affiliations:** 1 Instituto de Ciencias Agroalimentarias, Animales y Ambientales (ICA3), Universidad de O’Higgins, San Fernando, Chile; 2 Plant Morphogenesis and Biochemistry Laboratory, Federal University of Santa Catarina, Florianópolis, Santa Catarina, Brazil; 3 Faculty of Applied Ecology, Agricultural Sciences and Biotechnology University of Innland Norway, Blæstad, Norway

**Keywords:** abiotic stress, agriculture, GHG emissions, greenhouse gases, plant biotechnology

## Introduction

No part of the world is free from the severe impacts of climate change, which include rising temperatures, environmental degradation, and increased conflict. With agriculture being a significant contributor to greenhouse gas emissions, there is a pressing need for collective action and investment in agricultural bio-inputs. Technological advancements in this sector offer sustainable alternatives to traditional fertilizers and pesticides. The Research Topic “Mitigating agricultural greenhouse gas emissions through bio-inputs and innovative practices” compiles nine contributions focused on current agricultural strategies aimed at reducing these emissions.

## Reducing agricultural emissions through climate-smart practices

**Figure 1:**
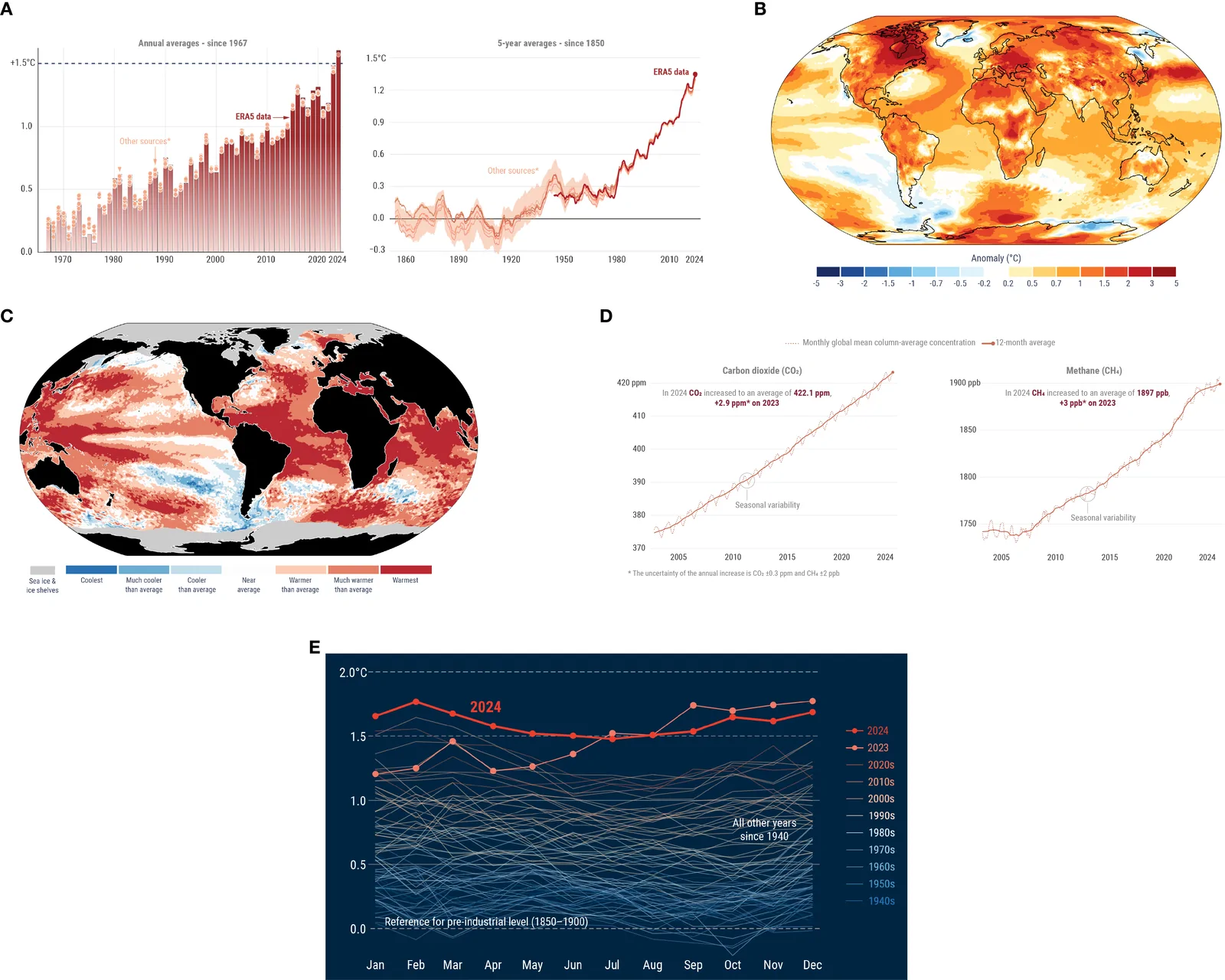
**(A)** Global surface air temperature increases above the 1850-1900 pre-industrial reference period, based on several global temperature datasets shown as annual averages since 1967 (left) and as 5-year averages since 1850 (right). **(B)** Surface air temperature anomalies for 2024 relative to the average for the 1991-2020 reference period. **(C)** Anomalies and extremes in sea surface temperature for 2024. Colour categories refer to the percentiles of the temperature distributions for the 1991-2020 reference period. The extreme ('coolest' and 'warmest') categories are based on rankings for the period 1979-2024. Values are calculated only for the ice-free oceans. **(D)** Monthly global atmospheric CO2 (left) and CH4 (right) column-averaged concentration derived from satellite data for 2003-2024 (dotted line) and 12-month average (solid line). **(E)** Global surface air temperature increase (°C) above the average for the pre-industrial reference period (1850-1900) for each month from January 1940 to December 2024, plotted as time series for each year. 2024 is shown as a thick red line and 2023 as a thick pink line, while other years are shown with thin lines and shaded according to the decade, from blue (1940s) to red (2020s). Data source: ERA5. Credit: C3S / ECMWF. Available at: https://climate.copernicus.eu/copernicus-2024-first-year-exceed-15degc-above-pre-industrial-level. ERA5 is the fifth generation European Centre for Medium-Range Weather Forecasts (ECMWF) atmospheric reanalysis of the global climate covering the period from January 1940 to present. All quoted temperature statistics covered in the global climate highlights are derived from ERA5. Source: Credit: ECMWF, Copernicus Climate Change Service (C3S).

Climate change threatens global food security and rural livelihoods, affecting hundreds of millions. While agriculture contributes 10-15% of global greenhouse gas emissions, including indirect emissions raises this to over 30%. Sustainable adaptations in agriculture are essential for mitigating climate change without compromising food security for a growing population. It is crucial to consider other agricultural outputs, such as ecosystem services, in the mitigation approach (Smith et al., 2007; Bellarby et al., 2008; Muller et al., 2011). Human activities, particularly modern agriculture, significantly contribute to greenhouse gas (GHG) emissions, which have risen since the industrial era. Major GHGs from agriculture include carbon dioxide (CO2), methane (CH4), and nitrous oxide (N2O), each with varying global warming potentials and atmospheric lifetimes. Agricultural soils account for approximately 15% of anthropogenic climate warming, predominantly through CO2 (74%), N2O (17%), and CH4 (9%) emissions. CH4 primarily arises from ruminant digestion and manure management, while N2O emissions result from soil microbial activities influenced by nitrogen inputs from fertilizers and manure (Cerri et al., 2007; Johnson et al., 2007; Gerber et al., 2013; Petersen et al., 2013; Hristov et al., 2015; Llonch et al., 2017; Abalos et al., 2022; Kopittke et al., 2024; Madjar et al., 2025). Agrifood systems are responsible for about one-third of global greenhouse gas (GHG) emissions, with agriculture contributing 49% of methane (CH4) and 63% of nitrous oxide (N2O) emissions in the European Union. Indirectly, agriculture raises GHG levels through land-use changes like deforestation. **Figure 1** summarizes current trends on Surface air and sea temperature anomalies in 2024. Mitigation strategies include changes in agronomic practices, animal diet modifications, manure treatment, and biological innovations. However, the effectiveness of these strategies varies based on farm diversity, resource availability, and policy support. Additionally, trade-offs between emission reduction, food security, and farmer livelihoods necessitate context-specific sustainable management approaches (Crippa et al., 2021; Foley et al., 2011; Madjar et al., 2025; Karki et al., 2025; Abalos et al., 2022; Xie et al., 2025; Rosenzweig and Tubiello, 2007; Wang et al., 2025). Agricultural lands constitute about 37% of the global land surface and are a major contributor to greenhouse gas (GHG) emissions, such as carbon dioxide (CO2), methane (CH4), and nitrous oxide (N2O), which significantly impact global warming. Key sources of these emissions include soil management, fertilizer usage, livestock practices, fossil fuel consumption, and land use changes. To mitigate GHG emissions, climate-smart practices like conservation tillage, cover crops, and efficient fertilizer use are recommended. Enhancing soil management to reduce disturbances and improving nitrogen use efficiency can further minimize carbon loss and fertilizer nitrogen application (Zaman et al., 2021; Yadav et al., 2024).

This Research Topic updates agricultural efforts and bio-input strategies aimed at reducing greenhouse gas emissions. A field experiment by Samphire et al. on an organic vegetable farm assessed emissions of nitrous oxide (N2O), methane (CH4), carbon dioxide (CO2), and potential ammonia (NH3) from soil when growing leeks or cabbages, with biodegradable plastic film mulch (PFM) and poultry manure or greenwaste compost. Results showed that, on average, yield increased by 26% with PFM, while potential NH3 emissions were 18% lower—43% when scaled to yield—in mulched compared to unmulched treatments. CH4 emissions remained largely unchanged. Furthermore, yield-scaled N2O emissions were 62% higher for mulched leeks but 56% lower for mulched cabbages, correlating with increased soil NO3- content in mulched leeks. The findings suggest that biodegradable PFM may effectively reduce harmful gaseous N emissions in organic horticulture. Viana et al. examined the link between higher coffee quality, its bioactive chemical profile, and sustainable agricultural practices. The researchers found that better coffee quality is associated with regions that employ sustainable management. Notably, higher altitudes correlated with reduced levels of xanthine alkaloids, ferulic acid, and p-coumaric acid. Increased soil organic matter also related to lower concentrations of trigonelline, theophylline, caffeine, and ferulic acid. Additionally, higher soil organic matter and carbon stock were positively correlated with coffee quality. The study concluded that adopting sustainable practices in coffee production not only mitigates greenhouse gas emissions but also enhances coffee quality and value. Moretti et al. examined the effects of foliar inoculation with Azospirillium brasilense and phosphorus (P) application on off-season maize yield and nutrition under reduced nitrogen (N) fertilization in Brazil. Their findings indicated that the combined use of A. brasilense and foliar P enhanced leaf N and P levels, stalk diameter, grain count per ear, grain weight, and overall grain yield (GY). While GY was highest with full N application, a 25% reduction in N, combined with the treatments, maintained similar GYs and decreased N2O emissions. This approach optimizes nutrient use efficiency, supports productivity, and reduces environmental impact, making it a viable strategy for sustainable off-season maize cultivation in Brazil. Rosello et al. assessed safer alternatives to chemicals for disease management and food shelf life preservation, finding that essential oils (EOs) effectively inhibited the growth of various phytopathogenic fungi. Notably, T. serpyllum biofilm showed promise in rice storage and controlled Fusarium oxysporum f. sp. lycopersici, enhancing the shelf life of tomatoes. The authors propose using these EOs as biofilms for safe food conservation, offering an alternative to synthetic products. Li et al. isolated two N2O-reducing bacterial strains: Enterobacter sp. (nosZ clade I) and Pseudomonas sp. (nosZ clade II). Their performance was evaluated under various environmental conditions regarding N2O emissions from agricultural soils with different fertilization strategies. Growth and N2O-reducing activity were significantly influenced by pH, oxygen levels, nitrate concentration, and carbon source. Soil microcosm experiments showed that fertilization type strongly affected N2O emissions, with mixed organic-inorganic treatments yielding the highest emissions. Inoculating with the nosZII strain markedly reduced emissions in soils with inorganic or combined fertilizers, while nosZI strain generally increased emissions except under mixed fertilization. These results emphasize the necessity of selecting microbial strains based on functional capacity and environmental tolerance, paving the way for targeted strategies to mitigate N2O emissions for sustainable agriculture. Oliveira et al. examine how marine and terrestrial biostimulant elicitors improve plant tolerance to cold stress. They identify macro- and microalgae as key suppliers of plant elicitors in aquatic settings, providing extractable molecules such as polysaccharides, polyamines, polyphenols, and amino acids that enhance plant defense mechanisms. Terrestrial plants are also noted for their potential to yield biostimulant compounds. Altshuler et al. developed an AI-driven model to predict the effectiveness of feed additives, specifically allicin-based essential oil (Allimax), in reducing methane emissions from Holstein cows. Validating the model across ten commercial farms over three months, with 339 cows, revealed two scenarios: a naive, uniform application of the additive and an optimized precision agriculture approach targeting farms predicted to benefit most. The results showed high predictive accuracy of the model and indicated that the optimized scenario resulted in greater methane reductions than the naive approach, highlighting the advantages of using rumen microbiome genetics in feed additive application. Zhang et al. found that a single application of controlled-release fertilizer (CRF) at the seedling stage boosts grain yield and reduces methane emissions in coastal saline soils. A two-year field study assessed two rice cultivars, Nanjing 5718 and Yongyou 4953, under four nitrogen treatments. The N3 treatment (50% 120-day CRF + 50% basal urea) significantly increased grain yield by 10.2 to 12.9% compared to conventional split urea (N1), while the N2 treatment (50% 80-day CRF + 50% basal urea) decreased yield by 11.9 to 13.0%. Methane emissions were highest with N1, decreasing with N2 and N3; N2 reduced peak CH4 flux by 18.9% and total emissions by 20.4%, while N3 led to reductions of 6.8% and 7.7%, respectively. Additionally, N3 improved root growth metrics, including root length and surface area. Huang et al. assessed agricultural plastic film carbon emissions in China, identifying agricultural machinery power (MACH) and plastic film usage (PFU) as primary factors, though their significance varies by region. They found that exceeding certain input levels leads to sharp emission increases, particularly in high-mechanization, intensive film use, and water-rich conditions. The study highlights the need for low-carbon agricultural strategies tailored to regional specifics to optimize resource utilization and minimize environmental effects.
